# A role for plasma membrane Ca^2+^ ATPases in regulation of cellular Ca^2+^ homeostasis by sphingosine kinase-1

**DOI:** 10.1007/s00424-024-03027-7

**Published:** 2024-10-11

**Authors:** Luisa Michelle Volk, Jan-Erik Bruun, Sandra Trautmann, Dominique Thomas, Stephanie Schwalm, Josef Pfeilschifter, Dagmar Meyer zu Heringdorf

**Affiliations:** 1 Institut Für Allgemeine Pharmakologie Und Toxikologie, Goethe-Universität Frankfurt, Universitätsklinikum, Frankfurt am Main, Germany; 2 Institut Für Klinische Pharmakologie, Goethe-Universität Frankfurt, Universitätsklinikum, Theodor-Stern-Kai 7, 60590 Frankfurt am Main, Germany; 3 Fraunhofer Institute for Translational Medicine and Pharmacology ITMP, Theodor-Stern-Kai 7, 60590 Frankfurt am Main, Germany

**Keywords:** Basigin, Ca^2+^ signaling, Plasma membrane Ca^2+^ ATPase, Sphingosine kinase, Sphingosine-1-phosphate, Sphingosine-1-phosphate lyase

## Abstract

Sphingosine-1-phosphate (S1P) is a ubiquitous lipid mediator, acting via specific G-protein-coupled receptors (GPCR) and intracellularly. Previous work has shown that deletion of S1P lyase caused a chronic elevation of cytosolic [Ca^2+^]_i_ and enhanced Ca^2+^ storage in mouse embryonic fibroblasts. Here, we studied the role of sphingosine kinase (SphK)-1 in Ca^2+^ signaling, using two independently generated EA.hy926 cell lines with stable knockdown of SphK1 (SphK1-KD1/2). Resting [Ca^2+^]_i_ and thapsigargin-induced [Ca^2+^]_i_ increases were reduced in both SphK1-KD1 and -KD2 cells. Agonist-induced [Ca^2+^]_i_ increases, measured in SphK1-KD1, were blunted. In the absence of extracellular Ca^2+^, thapsigargin-induced [Ca^2+^]_i_ increases declined rapidly, indicating enhanced removal of Ca^2+^ from the cytosol. In agreement, plasma membrane Ca^2+^ ATPase (PMCA)-1 and -4 and their auxiliary subunit, basigin, were strongly upregulated. Activation of S1P-GPCR by specific agonists or extracellular S1P did not rescue the effects of SphK1 knockdown, indicating that S1P-GPCR were not involved. Lipid measurements indicated that not only S1P but also dihydro-sphingosine, ceramides, and lactosylceramides were markedly depleted in SphK1-KD2 cells. SphK2 and S1P lyase were upregulated, suggesting enhanced flux via the sphingolipid degradation pathway. Finally, histone acetylation was enhanced in SphK1-KD2 cells, and the histone deacetylase inhibitor, vorinostat, induced upregulation of PMCA1 and basigin on mRNA and protein levels in EA.hy926 cells. These data show for the first time a transcriptional regulation of PMCA1 and basigin by S1P metabolism. It is concluded that SphK1 knockdown in EA.hy926 cells caused long-term alterations in cellular Ca^2+^ homeostasis by upregulating PMCA via increased histone acetylation.

## Introduction

Sphingosine-1-phosphate (S1P) is a bioactive lipid that regulates diverse cellular processes, including cell survival, proliferation, differentiation, and migration [6]. S1P is produced from sphingosine by the action of sphingosine kinases (SphK), SphK1 and SphK2. SphK1 is predominantly localized in the cytosol and can translocate to the plasma membrane upon activation, whereas SphK2 is found in nucleus, cytosol, mitochondria, and endoplasmic reticulum (ER) [34, 10]. The degradation of S1P occurs through reversible dephosphorylation back to sphingosine or irreversibly via S1P lyase (SGPL1), which represents the sole exit point of sphingolipid metabolism [36]. Extracellularly, S1P exerts its effects through five G-protein coupled receptors (GPCR), designated S1P_1-5_, which differentially couple to G_i_, G_q_, and G_12/13_ proteins. These receptors regulate for example lymphocyte trafficking, angiogenesis, vascular barrier function, and inflammation [6, 4, 30]. However, S1P also has intracellular functions independent of these receptors, which remain incompletely understood [41]. Notably, nuclear S1P produced by SphK2 interacted with histone deacetylases (HDAC)-1 and -2, inhibiting their enzymatic activity and thus contributing to the regulation of epigenetic mechanisms [16]. Other reported intracellular actions comprise for example stabilization of telomerase reverse transcriptase, or regulation of mitochondrial respiration via interaction with prohibitin-2, among others [42, 32].

Intracellular Ca^2+^ is a universal and versatile signal that is essential for cellular function [3]. The extensive Ca^2+^ signaling toolkit provides mechanisms for cell type-specific generation of spacial and temporal Ca^2+^ signals, which comprise “on” and “off” mechanisms [3]. In non-excitable cells, the key regulators that maintain low intracellular Ca^2+^ concentrations ([Ca^2+^]_i_) are plasma membrane Ca^2+^ ATPase (PMCA), which exports Ca^2+^ from the cytosol to the extracellular space, sarco-/endoplasmic reticulum Ca^2+^ ATPase (SERCA), which pumps Ca^2+^ from the cytosol into the ER, and secretory pathway Ca^2+^ ATPase (SPCA) [46, 45, 47]. In mammals, there are four isoforms of PMCA, of which PMCA1 and 4 are ubiquitously expressed, whereas PMCA2 and 3 are predominantly expressed in sensory cells and neurons [47, 40]. Interestingly, genome-wide association studies have associated the ATP2B1 gene, encoding PMCA1, with hypertension (for review, see [40]). Studies in mice with knockout of ATP2B1 in vascular smooth muscle cells showed that these mice had elevated systolic blood pressure and enhanced phenylephrine-induced vasoconstriction, most likely due to the increased [Ca^2+^]_i_ that was measured in cultured vascular smooth muscle cells of these mice [23]. Furthermore, heterozygous deletion of ATP2B1 proved that decreased PMCA1 activity promoted vascular remodeling in mice, which then developed hypertension at higher age [25]. Additionally, gene polymorphisms in ATP2B1 have been linked to other cardiovascular diseases such as coronary artery disease, myocardial infarction, and coronary artery calcifications in chronic kidney disease (reviewed in [40]). Also PMCA4 is widely expressed in the cardiovascular system, where it suppressed NO production and angiogenesis (reviewed in [40]). Furthermore, mutations in the ATP2B4 gene, encoding PMCA4, were protected against severe forms of malaria [43].

The influence of S1P on cellular Ca^2+^ homeostasis is highly complex. Early studies suggested that S1P was able to release Ca^2+^ directly from the ER [12] and probably acted as a second messenger mediating agonist-induced [Ca^2+^]_i_ increases [27]. Later, these studies were questioned by the discovery of the G-protein-coupled S1P receptors, which are able to induce [Ca^2+^]_i_ increases via classical G-protein pathways (for example [31]; reviewed in [48]). Nevertheless, by using the photolysis of caged S1P, we showed that intracellular S1P was able to mobilize Ca^2+^ from thapsigargin-sensitive stores independently of G-protein-coupled S1P receptors [49]. A potential involvement of the SphK/S1P pathway in agonist-induced [Ca^2+^]_i_ increases has been studied using the SphK inhibitors, N,N-dimethylsphingosine, and D,L-threo-dihydrosphingosine, which however are non-selective and unspecific [22, 9]. Further work has shown that S1P metabolism also can have a long-term influence on cellular Ca^2+^ homeostasis, for example, Sgpl1-deficient mouse embryonic fibroblasts had elevated basal cytosolic [Ca^2+^]_i_ and enhanced Ca^2+^ storage in ER and lysosomes [7, 50].

The aim of the present study was to re-investigate the putative role of the SphK1/S1P pathway in agonist-induced [Ca^2+^]_i_ increases by using SphK1-depleted cells. For this, we used two independently generated lines of EA.hy926 cells with stable, shRNA-mediated knockdown of SphK1, SphK1-KD1 [39], and SphK1-KD2 [51]. We show that depletion of SphK1 caused a marked decrease in basal cytosolic [Ca^2+^]_i_ and attenuated the overall [Ca^2+^]_i_ response to thapsigargin in both cell lines. Analysis of SphK1-KD2 cells revealed that this phenotype went along with a > 20-fold upregulation of PMCA1 protein expression, which was not due to signaling of S1P-GPCR but rather involved enhanced histone acetylation. In summary, by regulating PMCA, SphK1 exerts a profound long-term influence on cellular Ca^2+^ homeostasis and, in consequence, on Ca^2+^-dependent cellular functions.

## Materials and methods

### Materials

S1P, thapsigargin, K6PC-5, A971432, CYM5520, Gö6983, and vorinostat were purchased from Sigma-Aldrich (Taufkirchen, Germany). CYM5541 and CYM5442 were obtained from Cayman Chemical Company (Ann Arbor, USA). CYM50308 was purchased from Tocris Bioscience (Bristol, UK). Gö6976 was obtained from Selleckchem (Houston, USA). All other materials were from previously described sources [52].

### Cell culture

EA.hy926 cells were cultured in RPMI-1640 medium (Gibco/Thermo Fisher Scientific) supplemented with 10% fetal calf serum, 100 U/ml penicillin G and 0.1 mg/ml streptomycin, 10 mM HEPES, and 1.5 µg/ml puromycin in a humidified atmosphere of 5% CO_2_ and 95% air at 37 °C. EA.hy926 SphK1 knockdown cell line 1 (SphK1-KD1) has been generated by Drs. Andrea Huwiler and Stephanie Schwalm [39], while SphK1 knockdown cell line 2 (SphK1-KD2) has been generated by Dr. Gergely Imre [51]. For microscopy, the cells were seeded onto poly-L-lysine-coated 8-well slides (μ-slide; ibidi GmbH, Martinsried, Germany). Before experiments, the cells were kept in serum-free medium overnight.

### [Ca^2+^]_i_ measurements

[Ca^2+^]_i_ was determined using the ratiometric dye fura-2 in a Hitachi F2500 spectrofluorometer as described before [5]. Briefly, monolayer of EA.hy926 cells were detached with trypsin, resuspended in Hank’s balanced salt solution (HBSS; 118 mM NaCl, 5 mM KCl, 1 mM CaCl_2_, 1 mM MgCl_2_, 5 mM glucose and 15 mM HEPES, pH 7.4) and loaded with 1 µM fura-2/AM for 1 h at room temperature. After washing the cells twice with HBSS, they were resuspended at a density of ∼ 1 × 10^6^ cells/ml. Thereafter, fura-2 fluorescence was monitored. The excitation switched between 340 and 380 nm while emission was recorded at 510 nm. [Ca^2+^]_i_ was calculated after determination of maximum and minimum fluorescence according to Grynkiewicz et al. [14]. For measurements in the absence of extracellular Ca^2+^, the cells were resuspended in Ca^2+^-free HBSS, 50 µM EGTA was added shortly before stimulation with agonists or thapsigargin, and 1 mM CaCl_2_ was readded thereafter.

### Inositol phosphate production

Inositol phosphate production was measured in cells labeled with [^3^H]inositol as described before [5]. Briefly, stimulation of [^3^H]inositol-labeled cells with agonists was performed for 20 min at 37 °C in the presence of LiCl. [^3^H]inositol phosphates were collected by ion exchange chromatography and quantified by liquid scintillation counting.

### Quantitative real-time PCR

RNA was isolated using RNeasy plus mini kit (QIAGEN, Hilden, Germany), quantified using a NanoDrop spectrophotometer (Thermo Fisher Scientific, Dreieich, Germany), and transcribed into cDNA using a RevertAid First Strand cDNA Synthesis Kit (Applied Biosystems/Thermo Fisher Scientific) according to the manufacturer’s instructions. Quantitative real-time PCR was performed with the Applied Biosystems Quant Studio 3 Real-Time PCR System (Applied Biosystems/Thermo Fisher Scientific). The following TaqMan probes were used: ATP2B1 (Hs01001490_m1), ATP2B4 (Hs00608066_m1), S1PR1 (Hs01922614_s1), S1PR2 (ARRWEXF), S1PR3 (Hs00245464_s1), S1PR4 (Hs02330084_s1), and S1PR5 (Hs_00258220_s1), all labeled with FAM on the 5′ end. For the following probes, an individually designed TaqMan Array was used: 18S (Hs99999901_s1), SGPL1 (Hs00187407_m1), SPHK1 (Hs00184211_m1), SPHK2 (Hs00219999_m1), SPNS2 (Hs01390449_g1), ATP2A1 (Hs01092295_m1), ATP2A2 (Hs00544877_m1), ATP2A3 (Hs01024558_m1), ATP2B1 (Hs00155949_m1), ATP2B2 (Hs01090453_m1), ATP2B3 (Hs05016448_s1), ATP2B4 (Hs00608066_m1), ATP2C1 (Hs00995930_m1), and ATP2C2 (Hs00939492_m1). All PCR materials were obtained from ThermoFisher Scientific.

### Western blotting and antibodies

Cells grown near to confluence on 6-cm dishes were lysed; the proteins were separated by SDS gel electrophoresis and blotted onto polyvinylidene difluoride membranes. Anti-SphK1 (10,670–1-AP) and anti-SphK2 (17,096–1-AP) antibodies were from Proteintech (Manchester, UK). Anti-PMCA1 (ab190355) and anti-CD147 (ab212057) antibodies were from Abcam (Cambridge, UK). For PMCA4, the antibody (MA1-914) from Invitrogen (Carlsbad, California, USA) was used. Anti-SGPL1 antibody (AF5535) was from R&D systems (Wiesbaden, Germany). Anti-histone H3-acetyl-lysine-9 (H3K9ac; 9649S) antibody and anti-phosphoserine protein kinase C (PKC) substrate (#2261) were purchased from Cell Signaling Technology (Danvers, Massachusetts, USA). Anti-β-actin (A5441) was from Sigma-Aldrich Chemie GmbH (Taufkirchen, Germany) and anti-GAPDH (GTX100118) antibody was from Genetex (Irvine, California, USA). HRP-conjugated secondary antibodies were from GE Healthcare (Freiburg, Germany), and the enhanced chemiluminescence system was from Millipore Corporation (Billerica, MA, USA).

### Immunocytochemistry

EA.hy926 cells grown on 8-well slides were fixed with 4% paraformaldehyde for 1 h on ice, washed with phosphate-buffered saline (PBS), and permeabilized with 0.1% Triton X-100 in PBS for 5 min at room temperature. Between each of the following steps, the cells were washed with PBS. First, they were blocked with 5% milk in PBS for 1 h at room temperature. Then, they were stained with anti-PMCA1 antibody (1:100, ab190355, Abcam, Cambridge, UK), anti-PMCA4 antibody (1:50, MA1-914, Invitrogen, Carlsbad, USA), or anti-CD147 antibody (1:100, ab21205, Abcam, Cambridge, UK) for 1 h at room temperature, followed by AlexaFluor 488-conjugated anti-rabbit secondary antibody (1:1000) for 1 h at room temperature in the dark. Thereafter, cells were stained with DAPI (1 µg/ml) for 90 s at room temperature in the dark. Finally, confocal laser scanning microscopy was performed with a Zeiss LSM510 Meta system equipped with an inverted Observer Z1 microscope and a Plan-Apochromat 63x/1.4 oil immersion objective (Carl Zeiss MicroImaging GmbH, Göttingen, Germany). The following excitation (ex) laser lines and emission (em) filter sets were used: DAPI: ex 405 nm, em band pass 420–480 nm, AlexaFluor 488: ex: 488 nm, and em: long pass 505 nm.

### High-performance liquid chromatography tandem mass spectrometry

Concentrations of S1P, sphingosine, ceramides, glucosylceramides, and lactosylceramides were determined by high-performance liquid chromatography tandem mass spectrometry (LC–MS/MS) essentially as described before [52]. Divergently, the instrumental setup used here was composed of an Agilent Infinity II LC System (Agilent Technologies, Waldbronn, Germany) coupled to a mass spectrometer QTRAP6500 + (Sciex, Darmstadt, Germany).

### Data analysis and presentation

Averaged data are expressed as means ± SEM from the indicated number (*n*) of independent experiments. Statistical analysis was performed as indicated in the figure legends. Graphical presentations and statistical analyses were performed with GraphPad Prism (software version 9, GraphPad Software, San Diego, USA). Fluorescence images were edited with the ZEN software (Carl Zeiss MicroImaging GmbH, Göttingen, Germany). mRNA data were evaluated using the ∆∆Ct method, normalized to 18 s and expressed as fold of control cells. Western blot data were quantified using the iBright Analysis Software (ThermoFisher Scientific, Rockford, Illinois, USA), normalized to ß-actin or GAPDH as indicated, and expressed as fold of controls. For quantification of [Ca^2+^]_i_ increases, area under the curve (AUC) measurements were performed by integrating the area of thapsigargin-induced [Ca^2+^]_i_ above basal [Ca^2+^]_i_ at all time points up to 120 s by using GraphPad Prism.

## Results

**Fig. 1:**
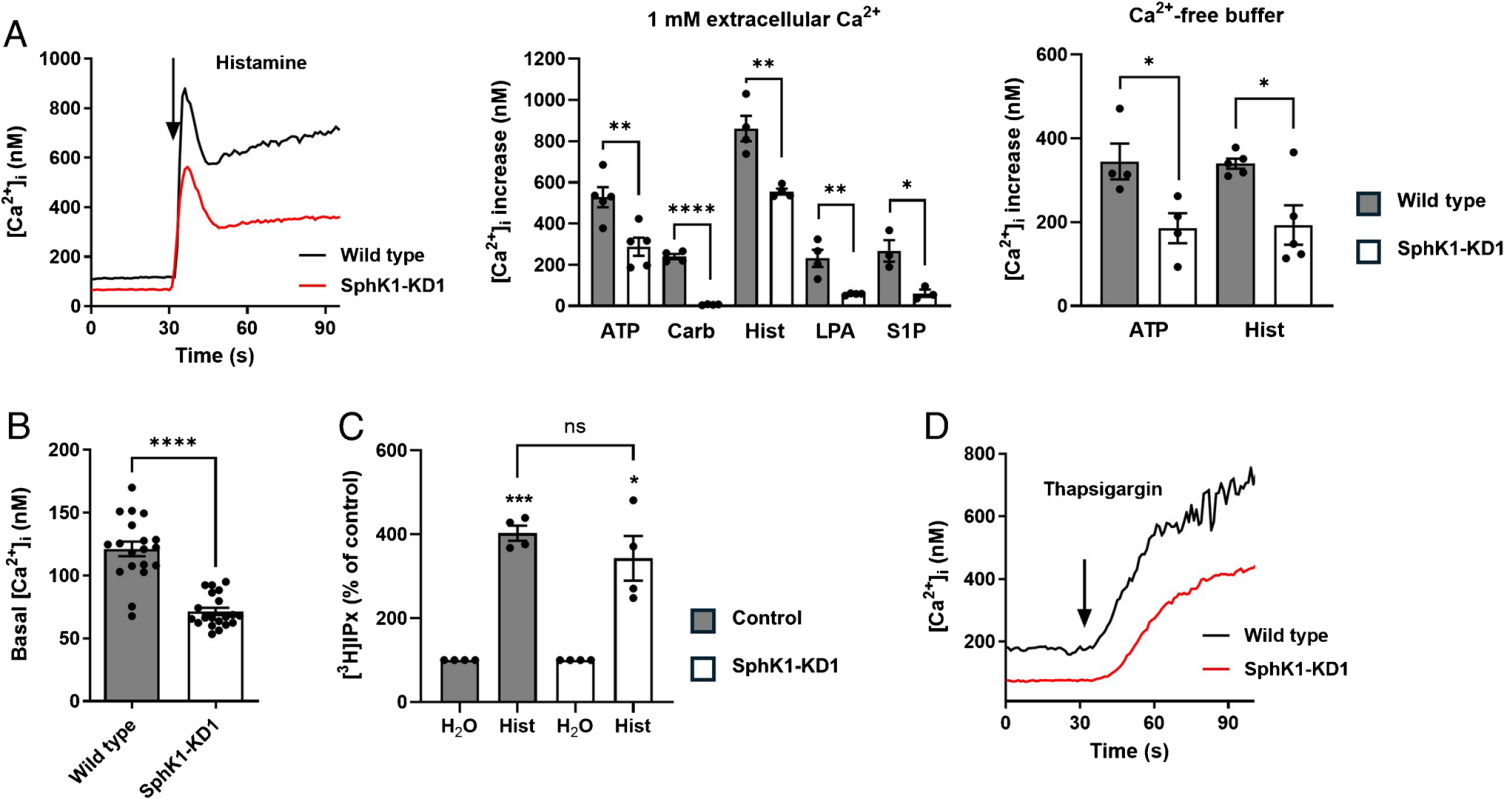
Ca^2+^ homeostasis in EA.hy926 cells with SphK1 knockdown, cell line 1 (SphK1-KD1). **A** Agonist-induced [Ca^2+^]_i_ increases in the presence or absence of 1 mM extracellular Ca^2+^. Shown is a representative time course of [Ca^2+^]_i_ after stimulation with histamine, and peak [Ca^2+^]_i_ increases after stimulation with 100 µM ATP, 100 µM carbachol (Carb), 100 µM histamine (Hist), 1 µM LPA, or 1 µM S1P (means ± SEM from *n* = 3–5 independent experiments for each agonist). **B** Basal [Ca^2+^]_i_ in the presence of 1 mM extracellular Ca^2+^ (data taken from the measurements shown in A; means ± SEM; *n* = 20). Data in **A** and **B** were analyzed by Student’s *t*-test (*p < 0.05; **p < 0.01; ****p < 0.0001). **C** Histamine-induced production of tritiated inositol phosphates ([^3^H]IPx) in cells labeled with [^3^H]inositol (means ± SEM; *n* = 4 independent experiments). Responses to histamine (100 µM) were analyzed for each cell line by one-sample *t*-test (*p < 0.05; ***p < 0.001) and compared between cell lines by Student’s *t*-test (n.s., not significant). **D** Representative time course of [Ca^2+^]_i_ after addition of thapsigargin (selected from ~ 20 measurements per cell line)

**Fig. 2:**
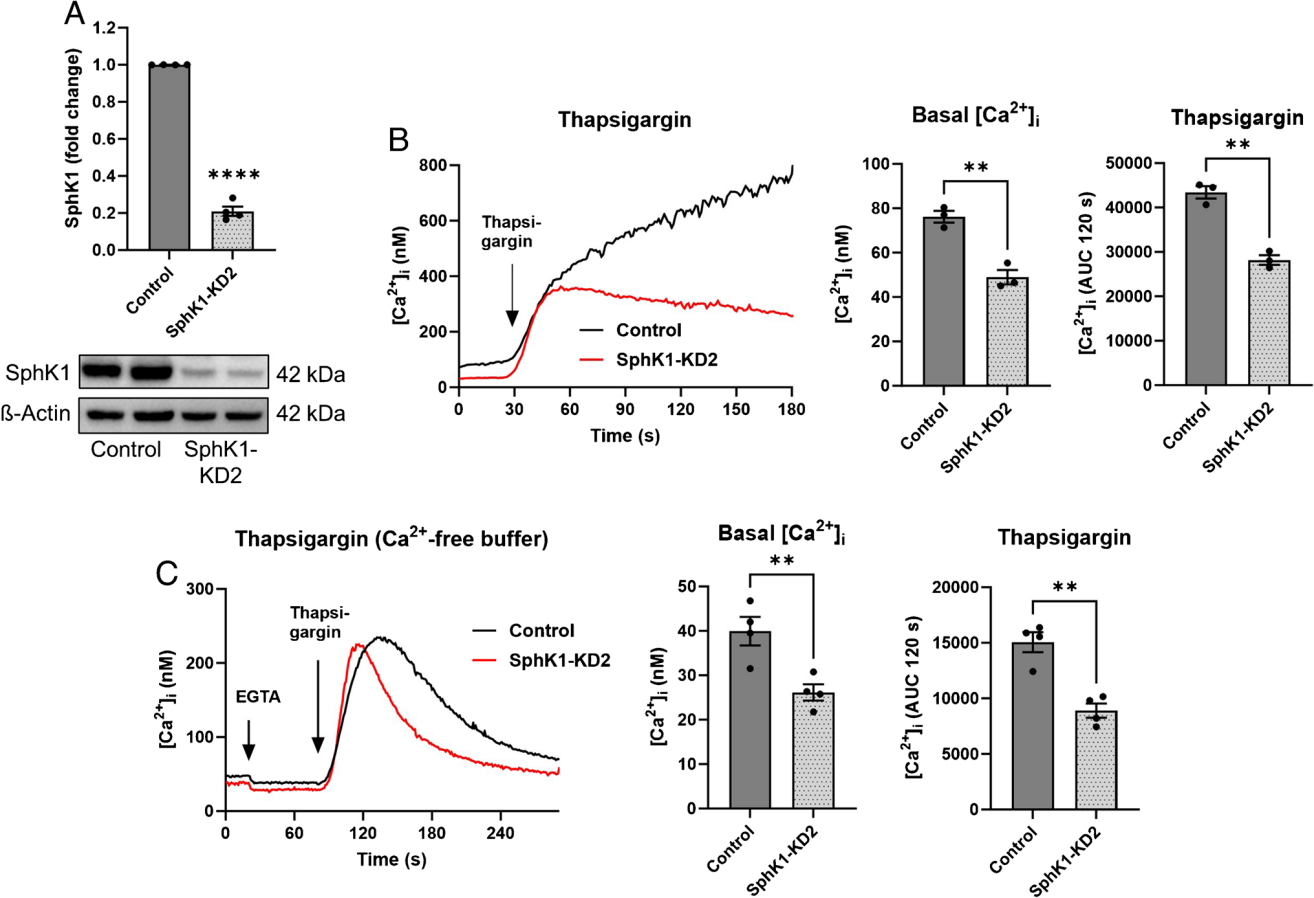
Ca^2+^ homeostasis in EA.hy926 cells with SphK1 knockdown, cell line 2 (SphK1-KD2). **A** Western blot analysis of SphK1 expression. Shown is a representative blot with duplicate samples, and the mean of four independent experiments performed in duplicates (means ± SEM; ****p < 0.0001 in one sample *t*-test). **B**, **C** Basal [Ca^2+^]_i_ levels and Ca^2+^ increases induced by 1 µM thapsigargin were measured in fura-2-loaded cells in the presence (**B**) or absence (**C**) of extracellular Ca^2+^. Shown are representative traces of [Ca^2+^]_i_ and means of 3 (**B**) or 4 (**C**) independent experiments. The response to thapsigargin was analyzed by measuring the [Ca^2+^]_i_ increase above baseline (area under the curve, AUC) for 120 s after addition of thapsigargin (AUC 120 s). In **C**, 50 µM EGTA was added to the Ca^2+^-free buffer ~ 1 min before addition of thapsigargin (means ± SEM; **p < 0.01 Student’s *t*-test)

With the aim to re-investigate the role of the SphK1/S1P pathway in Ca^2+^ signaling, we studied agonist-induced [Ca^2+^]_i_ increases in EA.hy926 SphK1-KD1 cells, in which shRNA-induced depletion of SphK1 reduced NO-mediated migration and tube formation [39]. As shown in Fig. 1A, peak [Ca^2+^]_i_ increases above baseline, induced by histamine, ATP, carbachol, lysophosphatidic acid (LPA), and external S1P, were significantly reduced in SphK1-KD1 cells. [Ca^2+^]_i_ increases by ATP and histamine were also analyzed in the absence of extracellular Ca^2+^ and reduced by ~ 40% also under this condition (Fig. 1A). Most importantly, we noticed that basal [Ca^2+^]_i_ was lower in SphK1-KD1 cells: it decreased from ~ 120 nM in wild type cells to ~ 70 nM in SphK1-KD1 cells (Fig. 1B). Furthermore, histamine-induced inositol phosphate production was not affected by the knockdown (Fig. 1C), but thapsigargin-induced [Ca^2+^]_i_ increases were reduced (Fig. 1D). Also, in the second EA.hy926 cell line with stable SphK1 depletion, SphK1-KD2 cells [51], basal [Ca^2+^]_i_ was markedly decreased by ~ 35% compared to control cells (Fig. 2A, B). Furthermore, the overall response to thapsigargin, measured as the area under the curve for 120 s of the [Ca^2+^]_i_ increase, was reduced by ~ 35% (Fig. 2B). Even in the absence of extracellular Ca^2+^, basal [Ca^2+^]_i_ was ~ 35% lower in SphK1-KD2 cells, and the response to thapsigargin was reduced by almost half (Fig. 2C). Importantly, thapsigargin-induced [Ca^2+^]_i_ increases declined much faster in SphK1-depleted cells, indicating that Ca^2+^ that was released from thapsigargin-sensitive stores was rapidly removed from the cytosol (Fig. 2C). These results demonstrate a profound long-term alteration of Ca^2+^ homeostasis in SphK1-depleted EA.hy926 cells.

**Fig. 3:**
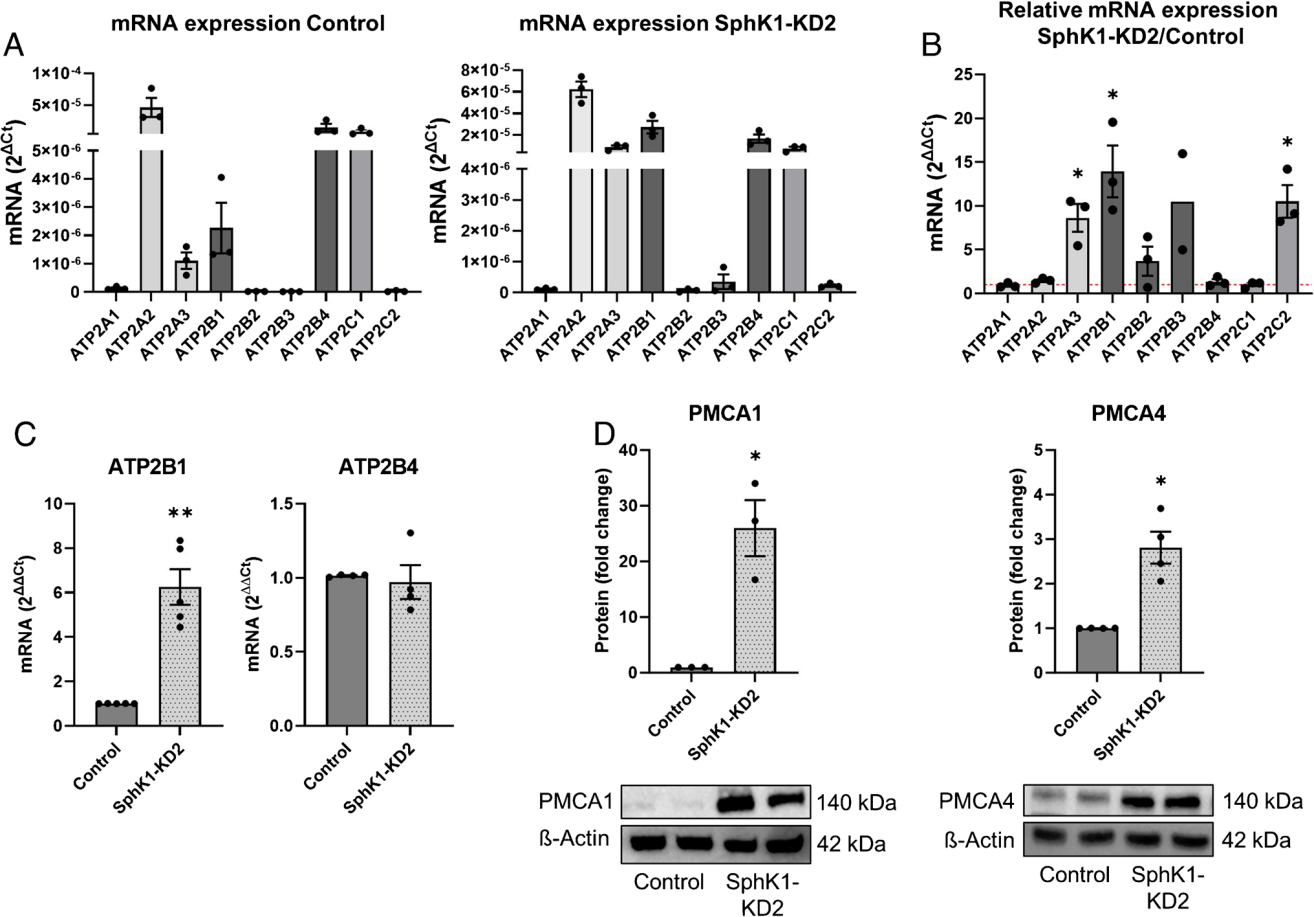
Expression of Ca^2+^-ATPases in SphK1-KD2 cells. **A**, **B** mRNA quantification by Taqman array (*n* = 3 independent experiments). **A** mRNA expression in control and SphK1-KD2 cells, shown as 2^ΔCt^. **B** mRNA expression in SphK1-KD2 relative to control cells, shown as 2^ΔΔCt^. **C** mRNA levels of ATP2B1 and ATP2B4: confirmation of Taqman array data by independent quantitative real-time PCR (*n* = 4). **D** Western blot analysis of PMCA1 and PMCA4 expression. Shown are representative blots from experiments performed in duplicates, and quantification of 3–4 independent experiments each. **A**–**D** All data represent means ± SEM and were analyzed by one-sample *t*-test (*p < 0.05; **p < 0.01)

**Fig. 4:**
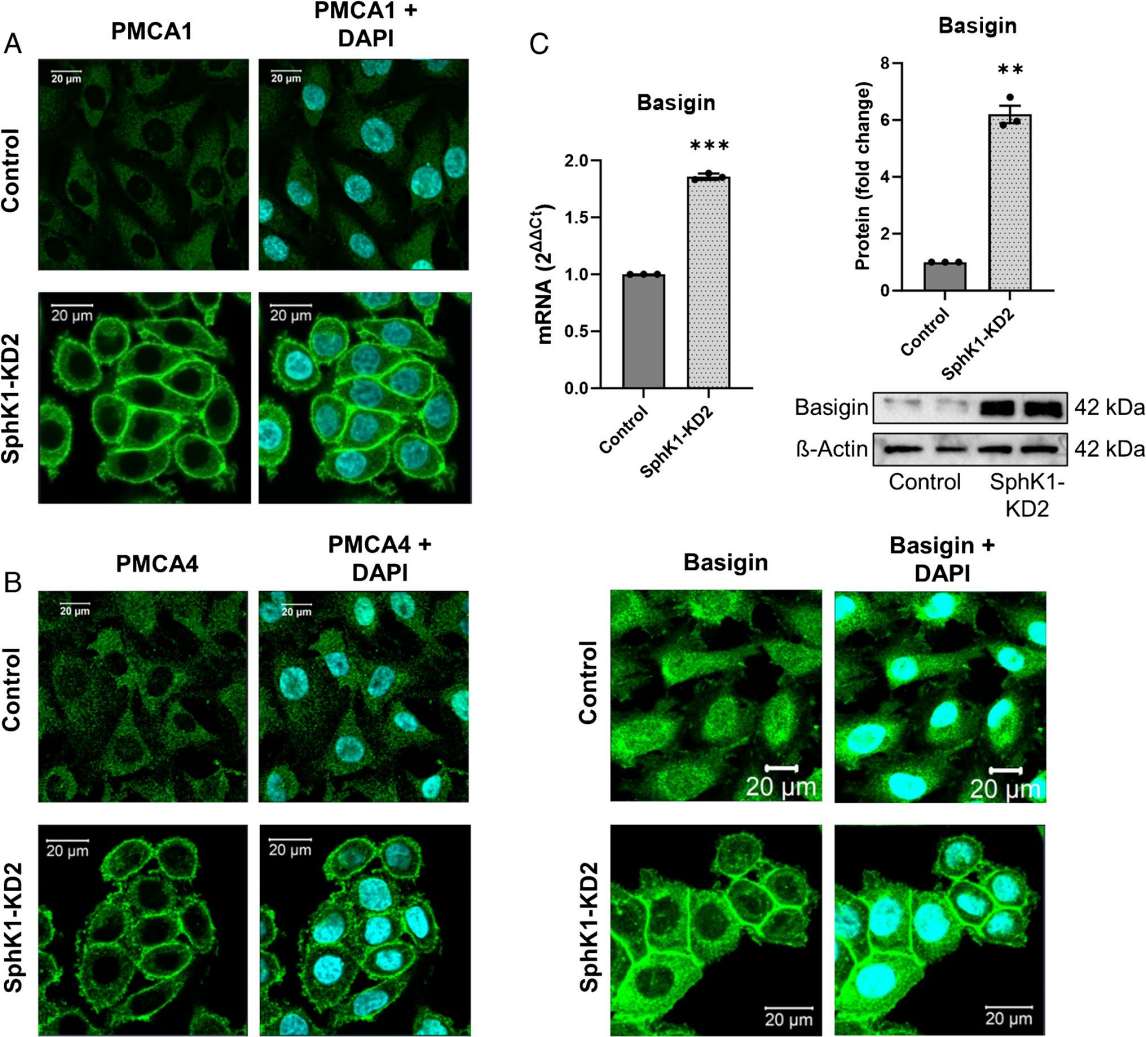
Intracellular localization of PMCA1 and 4, and expression of its essential auxiliary subunit basigin. **A**–**C** Localization of PMCA1, PMCA4, and basigin was investigated by immunocytochemistry and confocal laser scanning microscopy. Shown are representative images (bars = 20 µm). **C** mRNA expression and protein levels of basigin were analyzed by quantitative real-time PCR and Western blotting, respectively. Shown are values from three independent experiments (means ± SEM; **p < 0.01; ***p < 0.001 in one sample *t*-test)

Resting cytosolic [Ca^2+^]_i_ is kept low by the combined activities of the Ca^2+^ ATPases, SERCA, PMCA, and SPCA. Therefore, we measured the mRNA expression of Ca^2+^ ATPases by Taqman array (Fig. 3A, B). The results show that EA.hy926 control cells express mainly ATP2A2 (encoding SERCA2), ATP2B1 and 4, (PMCA1 and 4), and ATP2C1 (SPCA1). ATP2A1, ATP2B2, ATP2B3, and ATP2C2 were not detected on mRNA level, in agreement with their restriction to specific cell types [45, 40]. Figure 3B shows the relative changes of these mRNAs in SphK1-KD2 cells. Most importantly, the strongly expressed ATP2B1 was further upregulated in SphK1-depleted cells, whereas the other ATP2B isoforms were not significantly altered (Fig. 3B). Independent real-time PCR data confirmed that ATP2B1 mRNA was increased by ~ sixfold, while ATP2B4 mRNA was not altered (Fig. 3C). Furthermore, ATP2A3 and ATP2C2, only weakly expressed or nearly absent in control cells, were upregulated in SphK1-KD2 cells, although ATP2C2 mRNA was still very low (Fig. 3B). Remarkably, on protein level, PMCA1 was strongly increased by more than 20-fold in SphK1-KD2 cells (Fig. 3D). Although not regulated on mRNA level, PMCA4 protein was upregulated by ~ threefold (Fig. 3D). Furthermore, using immunostaining and confocal laser scanning microscopy, we observed a marked staining of PMCA1 and PMCA4 at the plasma membrane of EA.hy926 SphK1-KD2 cells, while there was only a weak non-specific background staining in control cells (Fig. 4A, B). Basigin (also known as CD147) and neuroplastin are two proteins that interact with PMCA enzymes and are regarded as “auxiliary subunits” of PMCA complexes [37]. They are interchangeably required for stability of PMCA complexes and their transport to the plasma membrane [37]. Figure 4C shows that, in SphK1-KD2 cells, indeed, not only PMCA1 and 4 but also basigin was strongly upregulated, both on mRNA and protein levels. Western blotting showed that basigin was expressed as a double band at ~ 42 kDa and was increased by ~ sixfold in SphK1-depleted cells. Immunocytochemistry and confocal microscopy revealed that there was some perinuclear and nuclear basigin staining in control cells, with only little plasma membrane localization, while there was a marked staining of basigin at the plasma membrane in SphK1-KD2 cells (Fig. 4C). Taken together, the marked upregulation of PMCA1 and PMCA4, together with basigin, explains very well the rapid removal of cytosolic Ca^2+^ in the presence of the SERCA inhibitor, thapsigargin, and the low basal [Ca^2+^]_i_ in SphK1-depleted cells.

**Fig. 5:**
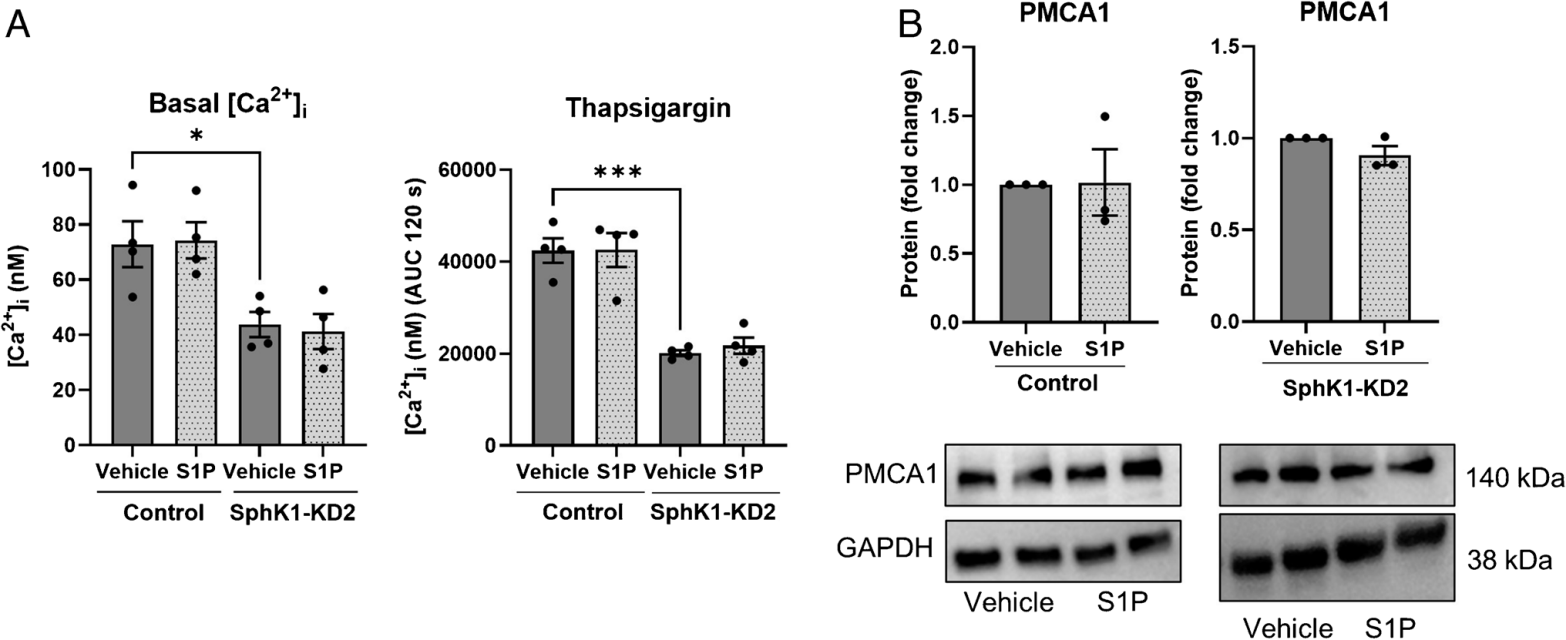
No influence of external treatment with S1P on Ca^2+^ homeostasis and PMCA1 expression. **A** Basal [Ca^2+^]_i_ and Ca^2+^ increases induced by 1 µM thapsigargin were analyzed after treatment with vehicle or 1 µM S1P for 16 h. Data are means of three independent experiments. Differences between the two cell types were analyzed by one-way ANOVA (means ± SEM; *p < 0.05; ***p < 0.001). **B** Protein expression of PMCA1 after treating control or SphK1-KD2 cells with vehicle or 1 µM S1P for 16 h, respectively. Shown are means from three independent experiments (means ± SEM)

**Fig. 6:**
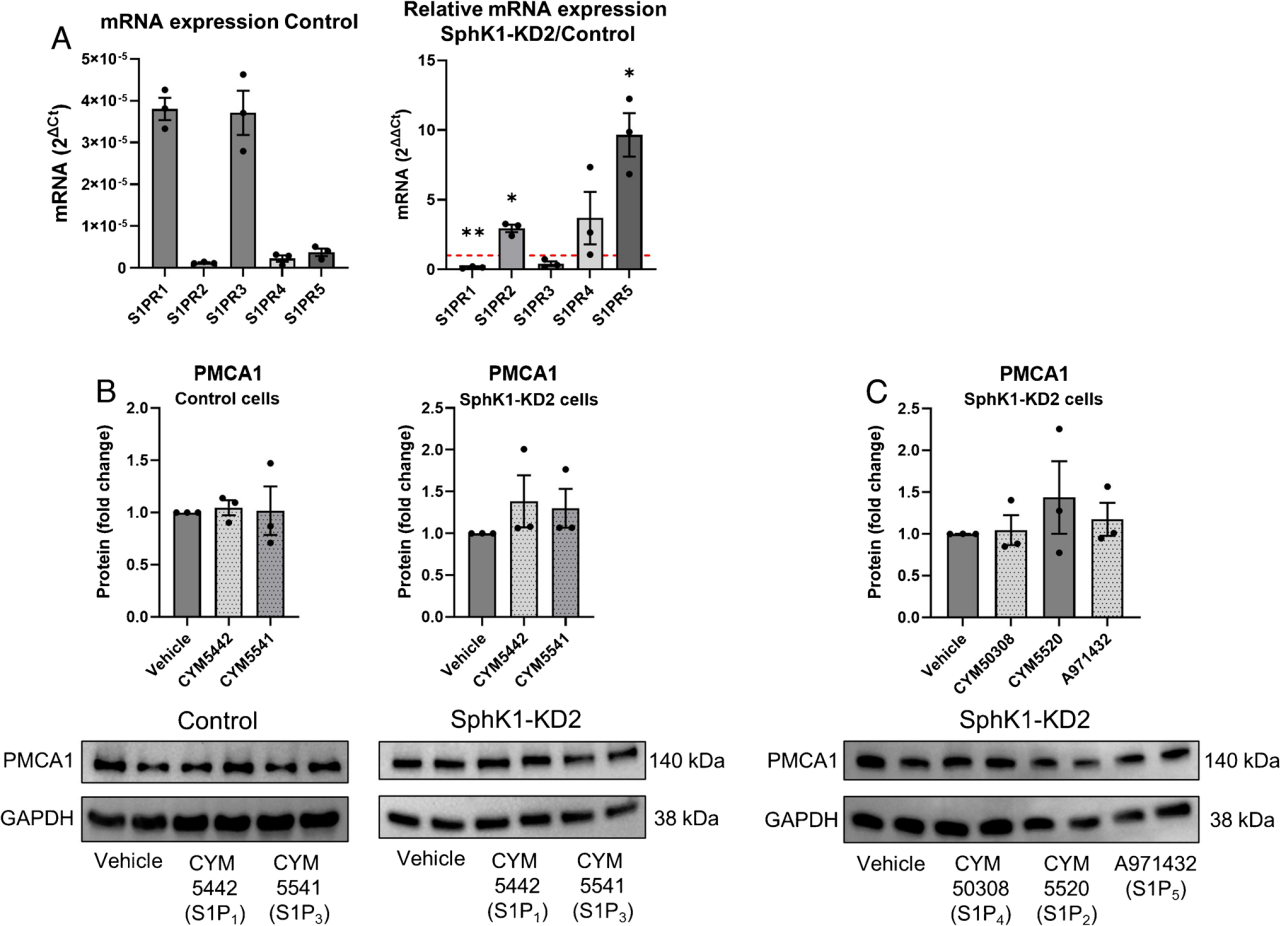
No influence of specific S1P receptor agonists on PMCA1 expression. **A** mRNA expression of S1PR1-S1PR5 in control cells (shown as 2^ΔCt^) and SphK1-KD2 cells (shown as 2^ΔΔCt^, relative to control cells). **B**, **C** Protein expression of PMCA1 after treating control or SphK1-KD2 cells with vehicle, CYM5442 (1 µM), CYM5541 (1 µM), CYM50308 (1 µM), CYM5520 (5 µM), or A971432 (1 µM) for 16 h, respectively. Shown are values from three independent experiments (means ± SEM; *p < 0.05; **p < 0.01 in one sample *t*-test)

Next, we followed the hypothesis that G-protein-coupled S1P receptors regulated PMCA expression, and depletion of SphK1 decreased autocrine S1P signaling. Therefore, we tested the influence of externally added S1P on Ca^2+^ homeostasis and PMCA1 expression in SphK1-KD2 cells. As shown in Fig. 5A, incubation with 1 µM S1P for 16 h did not have any effect on basal [Ca^2+^]_i_, nor did it alter the response to thapsigargin, in both control and knockdown cells. In agreement, PMCA1 protein expression was unaltered by external S1P (Fig. 5B). There are many examples for antagonistic signaling of S1P receptor subtypes [4]. Therefore, it was possible that individual S1P receptor subtypes modulated PMCA1 expression in an opposing manner. Thus, we also tested the influence of selective S1P receptor agonists on PMCA1 expression (Fig. 6). Quantitative real-time PCR measurements revealed that S1P_1_ and S1P_3_ mRNAs were clearly the most abundant S1P receptor transcripts in control cells and that S1PR2 and S1PR5 were upregulated in SphK1-KD2 cells, while S1PR1 was downregulated (Fig. 6A). Of note, neither CYM5442 (S1P_1_) nor CYM5541 (S1P_3_) altered PMCA1 protein expression in control or SphK1-KD2 cells (Fig. 6B). Furthermore, neither CYM5520 (S1P_2_), CYM50308 (S1P_4_), nor A971432 (S1P_5_) had an influence on PMCA1 in SphK1-KD2 cells (Fig. 6C). Taken together, the data suggest that SphK1 knockdown modulated PMCA1 expression independently of S1P-GPCR.

**Fig. 7:**
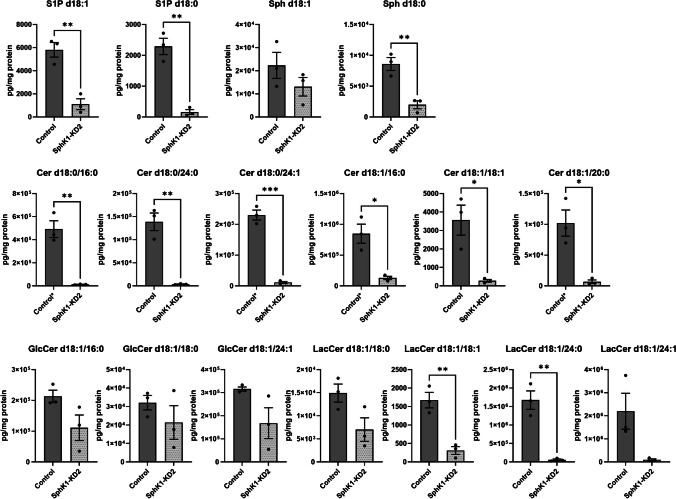
Sphingolipid concentrations in control and SphK1-KD2 cells. S1P, sphingosine (Sph), ceramides (Cer), glucosylceramides (GlcCer), and lactosylceramides (LacCer) were measured by LC–MS/MS in control and SphK1-KD2 cells. Shown are values from three independent experiments (means ± SEM; *p < 0.05; **p < 0.01; ***p < 0.001 in Student’s *t*-test). Control*: concentrations of these ceramides were above the upper limit of quantification in control cells, but not in SphK1-KD2 cells. After critical evaluation, these values were considered usable because of good reproducibility and the large difference between control and SphK1-KD2 cells; however, they have to be regarded as semi-quantitative

**Fig. 8:**
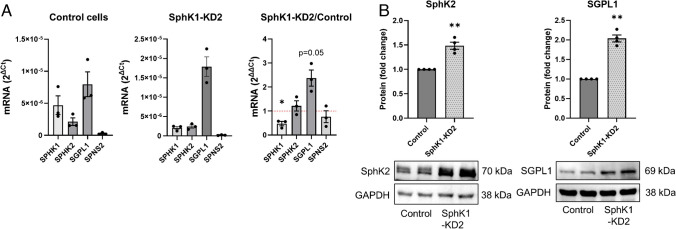
Expression of SphK, SGPL1, and SPNS2. **A** mRNA expression was analyzed by Taqman array in control and SphK1-KD2 cells (*n* = 3 independent experiments; *p < 0.05). **B** Protein expression of SphK2 and SGPL1. Shown are representative blots and quantifications of four independent experiments each (means ± SEM; **p < 0.01 in one sample *t*-test)

To address the question how SphK1 depletion altered sphingolipid concentrations in EA.hy926 cells, we analyzed the cells by LC–MS/MS. As expected, concentrations of S1P d18:1 and d18:0 were strongly reduced in SphK1-KD2 cells compared to control cells (Fig. 7). Interestingly, sphingosine d18:1 and d18:0 were not increased; sphingosine d18:0 (dihydro-sphingosine) was even significantly reduced. Intriguingly, all measured ceramides (d18:0/16:0, d18:0/24:0, d18:0/24:1, d18:1/16:0, d18:1/18:1, d18:1/20:0) were highly decreased in SphK1 knockdown cells (Fig. 7). In addition, certain lactosylceramides (d18:1/18:1, d18:1/24:0) were also strongly depleted (Fig. 7). Thus, despite the knockdown of SphK1, sphingolipids upstream of SphK did not accumulate, but in contrast were widely decreased. These data suggested that in the absence of SphK1, sphingolipid catabolism via SphK2 and SGPL1 led to a depletion of cellular sphingolipids in EA.hy926 cells. Taqman array data revealed that SPHK1 mRNA was reduced, in agreement with its knockdown, but SPHK2 mRNA was unaltered (Fig. 8A). The increase in SGPL1 mRNA was not significant (*p* = 0.05), while SPNS2 was nearly absent (Fig. 8A). However, Western blot analysis proved that both SphK2 and SGPL1 were upregulated on protein level (Fig. 8B). SphK2 appeared in two bands, likely splice variants [53], and was upregulated by ~ 1.5-fold, while SGPL1 was elevated by twofold (Fig. 8B). In summary, knockdown of SphK1 went along with upregulation of SphK2 and SGPL1 in EA.hy926 cells, leading to depletion of a wide range of sphingolipids. It remains unknown why the strong decrease particularly in S1P and ceramides obviously did not upregulate de novo sphingolipid synthesis.

**Fig. 9:**
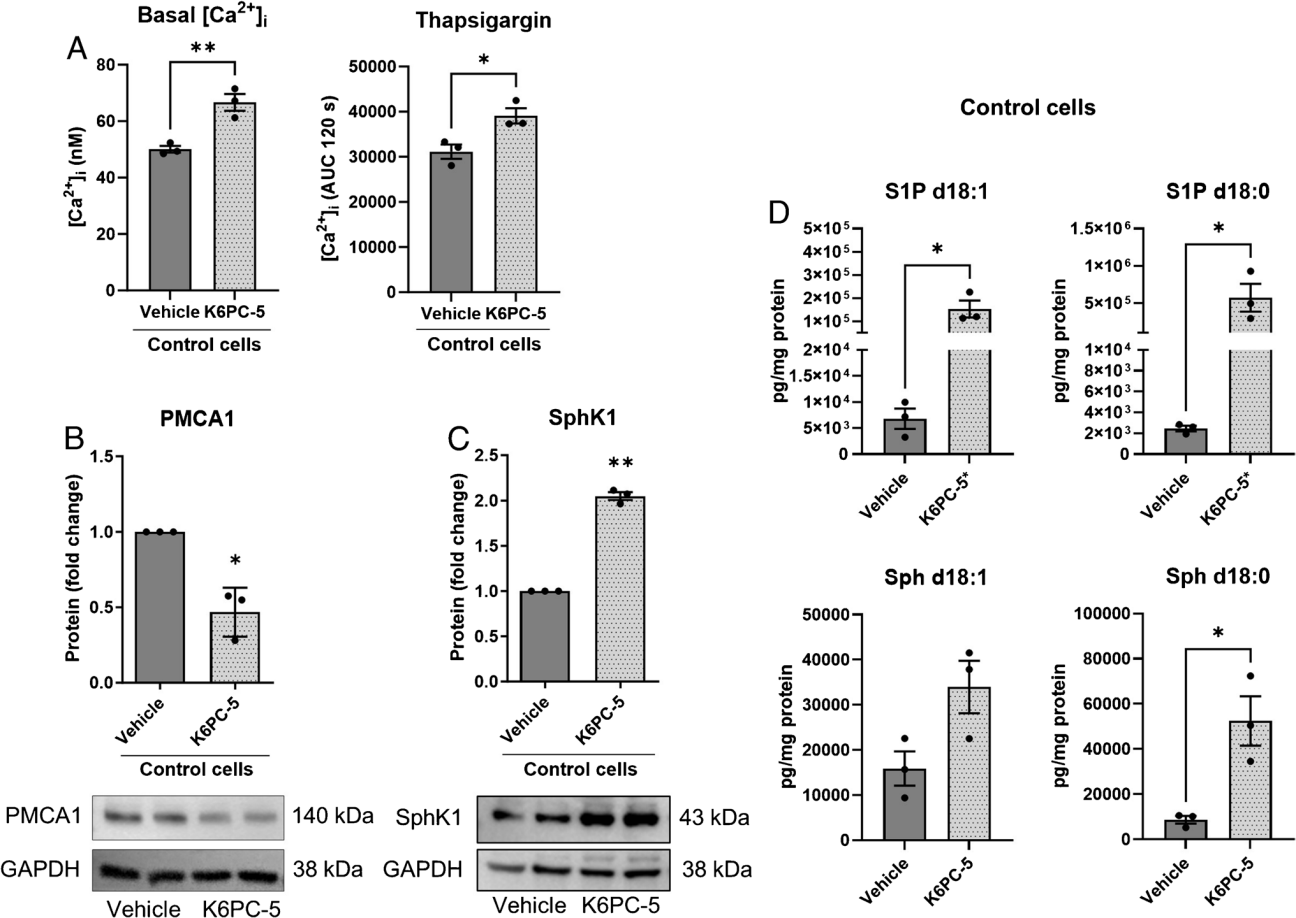
Effects of the SphK1 activator, K6PC-5, in EA.hy926 control cells. **A** Basal [Ca^2+^]_i_ and [Ca^2+^]_i_ increases induced by 1 µM thapsigargin were analyzed after treatment with vehicle or 50 µM K6PC-5 for 48 h. Data are means of three independent experiments. Statistical analysis was performed by Student’s *t*-test (means ± SEM; *n* = 3; *p < 0.05; **p < 0.01). **B**, **C** Protein expression of PMCA1 (**B**) and SphK1 (**C**) after treatment of control cells with vehicle or 50 µM K6PC-5 for 16 h. Shown are means from three independent experiments (means ± SEM; *n* = 3; *p < 0.05; **p < 0.01 in one sample *t*-test). **D** Concentrations of S1P and sphingosine (Sph) after treatment with vehicle or 50 µM K6PC-5 for 16 h. Values are from three independent experiments (means ± SEM; *p < 0.05 in Student’s *t*-test). KCPC-5*: after treatment with K6PC-5, concentrations of S1P d18:1 and S1P d18:0 were above the upper limit of quantification. After critical evaluation, these values were considered usable because of good reproducibility and the large difference between control and treated cells; however, they have to be regarded as semi-quantitative

To further address the role of SphK1 for PMCA expression and Ca^2+^ homeostasis, we treated control cells with the SphK1 activator, K6PC-5. Interestingly, treatment with 50 µM K6PC-5 for 48 h significantly increased basal [Ca^2+^]_i_ and augmented the response to thapsigargin in control cells (Fig. 9A). Furthermore, 50 µM K6PC-5 for 16 h reduced the expression of PMCA1 (Fig. 9B), which explains the effects on Ca^2+^ homeostasis. Interestingly, besides its known activity as stimulator of SphK1 activity, K6PC-5 also increased SphK1 protein expression in EA.hy926 control cells (Fig. 9C). In consequence, concentrations of S1P d18:1 were increased by > tenfold, and those of S1P d18:0 by > 100-fold by K6PC-5 (Fig. 9D). Sphingosine d18:0 was also significantly elevated (Fig. 9D). Taken together, it can be concluded that K6PC-5 caused effects in EA.hy926 control cells that were opposite to SphK1 knockdown.

**Fig. 10:**
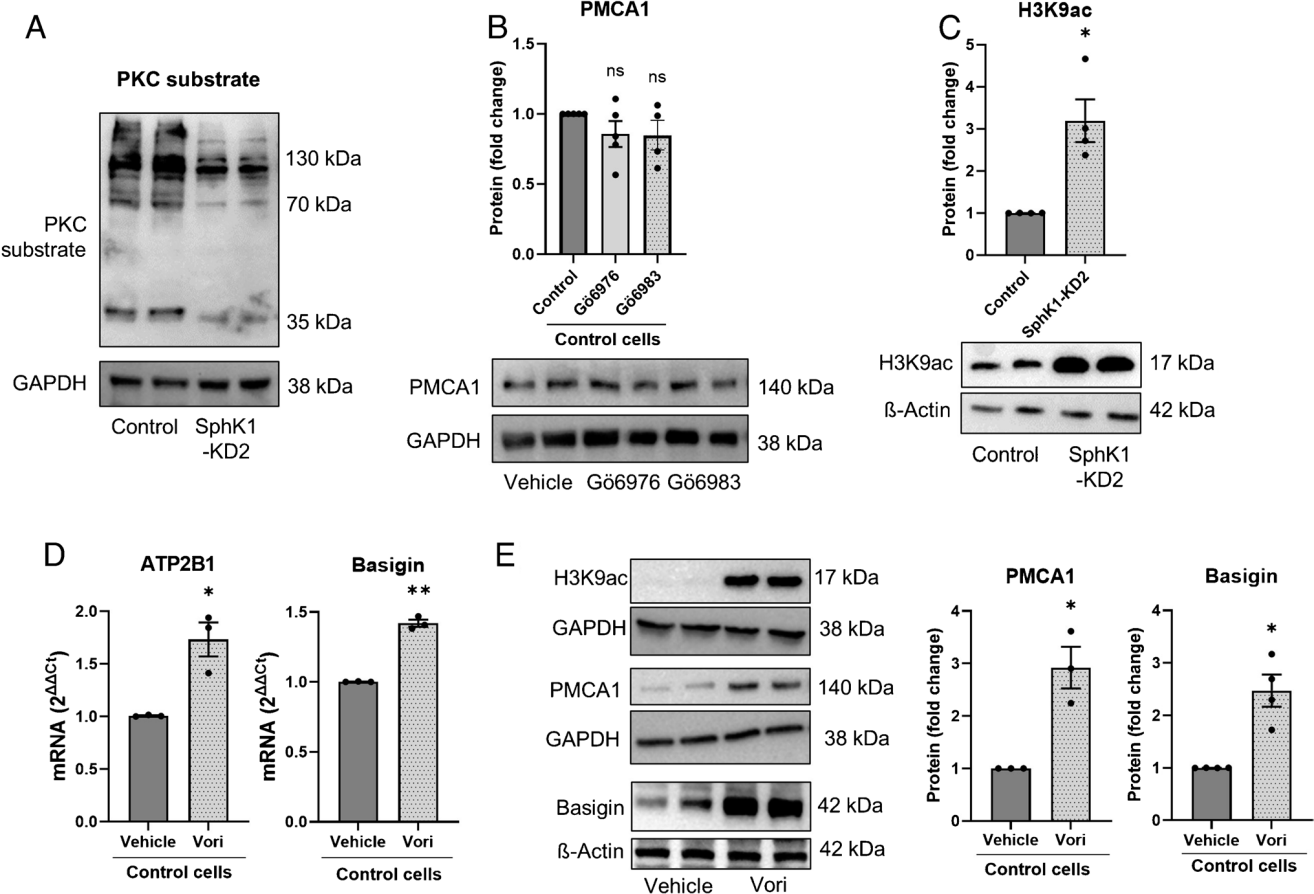
Mechanisms involved in transcriptional upregulation of PMCA1 in SphK1-KD2 cells. **A** Overall PKC activity was tested by Western blotting with an antibody directed at phosphorylated serine residues of PKC substrates in control and SphK1-KD2 cells. Shown is a representative experiment selected from 4 experiments with similar results. **B** Protein expression of PMCA1 in control cells after treatment with vehicle, 1 µM Gö6976 or 1 µM Gö6983 for 16 h (*n* = 4). **C** Analysis of H3K9 acetylation by Western blotting (*n* = 4). **D**, **E** Treatment of control EA.hy926 cells with 2 µM vorinostat (Vori) or vehicle for 24 h. **D** mRNA levels of ATP2B1 and basigin (*n* = 3). **E** H3K9 acetylation and protein expression of PMCA1 (*n* = 3) and basigin (*n* = 4). **B**, **C**, **E** Shown are representative blots and quantification of 3–4 independent experiments each (means ± SEM; n.s., not significant; *p < 0.05; **p < 0.01 in one sample *t*-test)

Only little is known about transcriptional regulation of PMCA1. Early studies had shown that PKC activation induced ATP2B1 mRNA transcription [24]. Since sphingosine is a known inhibitor of PKC [17], we hypothesized that the decrease in sphingosine d18:0 in EA.hy926 SphK1-KD cells might release PKC from inhibition and thereby upregulate ATP2B1 mRNA. However, as shown in Fig. 10A, PKC activity, detected with a phosphoserine-specific PKC substrate antibody, was clearly decreased in SphK1-KD2 cells. To address the possibility that the reduced PKC activity in SphK1-KD2 cells caused the increased expression of PMCA1, we treated control cells with either of the two PKC inhibitors, Gö6976 and Gö6983. While Gö6976 inhibits PKCα and PKCβ and a number of other kinases, Gö6983 has a higher specificity for PKCs and targets PKCα, β, δ, ε, η, and θ [1]. However, neither of the PKC inhibitors induced an upregulation of PMCA1 in EA.hy926 control cells (Fig. 10B). In conclusion, the reduced PKC activity in SphK1-KD2 cells is likely not the cause for PMCA1 upregulation, with the limitation that the inhibitors did not cover atypical PKC isoforms. On the contrary, the reduced PKC activity might be a consequence of the low resting [Ca^2+^]_i_ which dampened the activity of the classical Ca^2+^-dependent PKC isoforms.

**Fig. 11:**
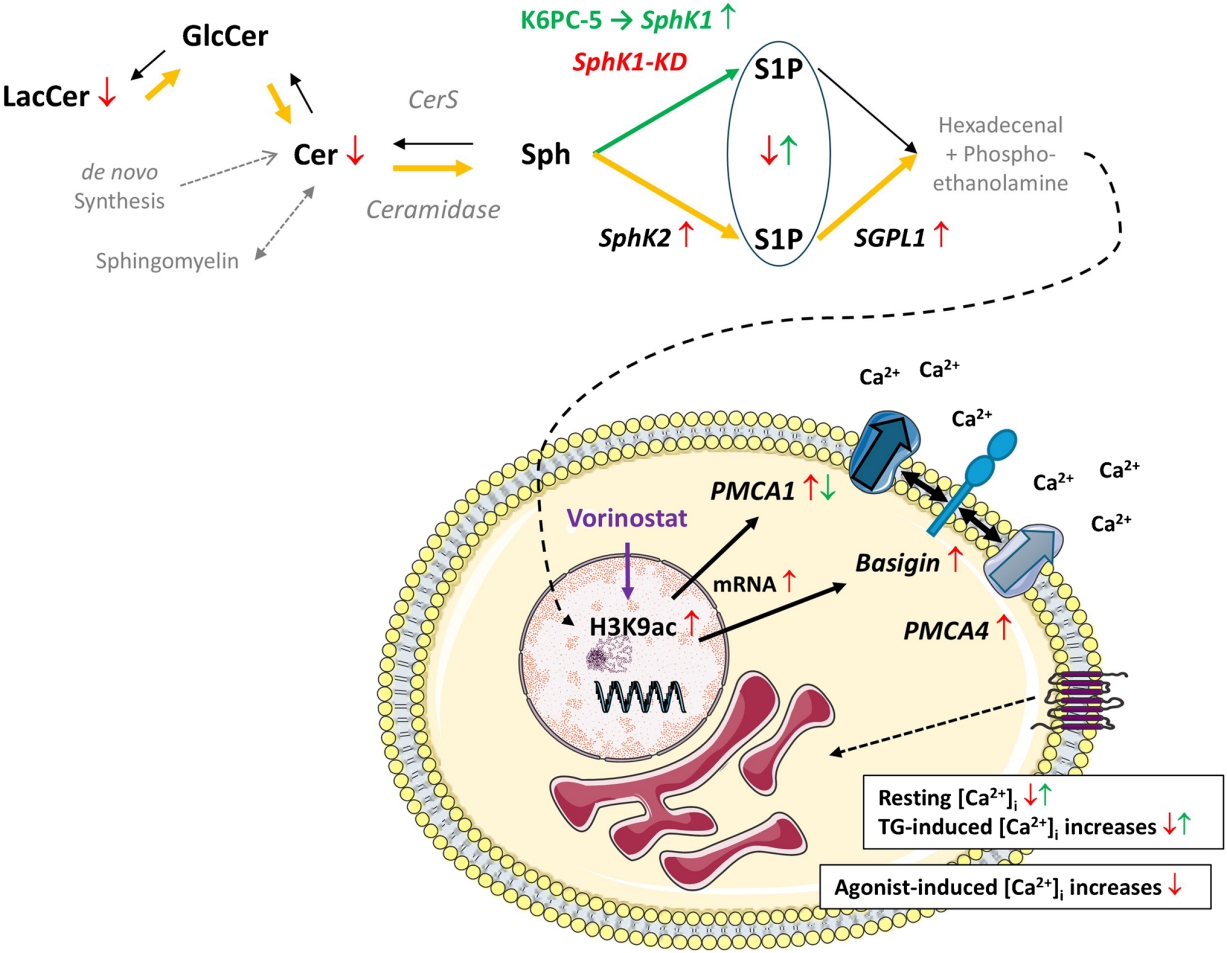
Graphical summary of the alterations in sphingolipid concentrations and Ca^2+^ homeostasis in EA.hy926 cells with stable knockdown of SphK1. Enzymes and lipids written in gray letters have not been addressed in the present study. The lipid analysis did not differentiate between S1P derived from SphK1 or SphK2. In SphK1-KD cells (red arrows), the concentrations of S1P, ceramides (Cer), and lactosylceramides (LacCer) were decreased. SphK2 and SGPL1 were upregulated, suggesting that there was enhanced flux via the sphingolipid degradation pathway (orange arrows). Conversely, the SphK1 activator, K6PC-5 (green arrows), caused an upregulation of SphK1 and increased S1P concentrations. SphK1-KD cells had reduced resting [Ca^2+^]_i_ and diminished [Ca^2+^]_i_ increases in response to thapsigargin (TG) and diverse GPCR agonists. In agreement, PMCA1 and its auxiliary subunit, basigin, were upregulated on mRNA and protein levels. Basigin is known to stabilize PMCA complexes and probably contributed to the increase in PMCA4 protein. Transcriptional regulation of PMCA1 and basigin was associated with enhanced histone acetylation (specifically, H3K9ac) and mimicked by the HDAC inhibitor, vorinostat. Conversely, K6PC-5 caused a decrease in PMCA1 expression and elevated both resting [Ca^2+^]_i_ and thapsigargin-induced [Ca^2+^]_i_ increases. Recently published data [11] show that hexadecenal, the product of SGPL1, induced HDAC inhibition, and we propose that this was the mechanism of enhanced histone acetylation in SphK1-KD cells

There are some reports that have shown that PMCA isoforms are differentially and in a cell-type-specific manner regulated by HDAC inhibitors (see for example [18, 33]). Since S1P metabolism may affect histone acetylation and thereby epigenetic regulation [16, 19, 54], we addressed the question whether histone acetylation was altered by knockdown of SphK1. Indeed, as shown in Fig. 10C, acetylation of histone-3 lysine-9 (H3K9) was clearly enhanced in SphK1-KD2 cells. Therefore, we further analyzed the influence of HDAC inhibition on expression of PMCA1 and basigin, using vorinostat (also known as suberoylanilide hydroxamic acid, SAHA). As shown in Fig. 10D, treatment with 2 µM vorinostat for 24 h caused a mild but significant upregulation of both ATP2B1 and basigin mRNA expression. Furthermore, vorinostat induced a strong increase in H3K9 acetylation and induced protein expression of both PMCA1 and basigin by ~ threefold and ~ 2.5-fold, respectively (Fig. 10E). These data support the hypothesis that transcription of ATP2B1 and basigin is induced by enhanced histone acetylation in EA.hy926 cells (Fig. 11).

## Discussion

Both acute receptor-induced Ca^2+^ signaling and long-term alterations in cellular Ca^2+^ homeostasis have been attributed to intracellular S1P and its formation and degradation. For example, in embryonic fibroblasts from Sgpl1-deficient mice, resting [Ca^2+^]_i_ and Ca^2+^ storage were elevated, and agonist-induced [Ca^2+^]_i_ increases were accordingly enhanced [7, 19]. Also, in INS1E rat insulinoma cells, knockdown of Sgpl1 increased [Ca^2+^]_i_, measured with the Ca^2+^ sensor Case12, while Sgpl1 overexpression slightly reduced [Ca^2+^]_i_ [15]. In contrast, SphK/S1P signaling has been viewed as a potential second messenger pathway, mediating acute agonist-induced [Ca^2+^]_i_ increases (reviewed in [48]). However, the results of these early studies are questionable since the specificity of the applied SphK inhibitors was low [22, 9]. Therefore, we revisited here the role of SphK1 in GPCR-induced Ca^2+^ signaling. We show that shRNA-mediated knockdown of SphK1 in EA.hy926 cells caused a marked reduction in agonist-induced [Ca^2+^]_i_ increases, both in the absence and presence of extracellular Ca^2+^, while agonist-stimulated inositol phosphate production, exemplarily measured for histamine, was not altered. Although this observation was in line with SphK1/S1P serving as signal transduction pathway for Ca^2+^ mobilization, further results did not match: in both of our two lines of EA.hy926 cells with stable knockdown of SphK1, basal [Ca^2+^]_i_ and [Ca^2+^]_i_ increases by the SERCA inhibitor, thapsigargin, were reduced. Thus, we demonstrate that besides SGPL1, also SphK1 can have a profound long-term effect on cellular Ca^2+^ homeostasis. We furthermore conclude that a specific role for SphK1 in acutely transmitting [Ca^2+^]_i_ increases by GPCR agonists cannot be studied using this genetic model of SphK1 deficiency.

It is highly likely that more than 20-fold upregulation of PMCA1 protein in SphK1-KD2 cells, supported by the ~ threefold upregulation of PMCA4, is the causative factor for the alterations in Ca^2+^ homeostasis in EA.hy926 SphK1 knockdown cells. In particular, the rapid clearing of cytosolic Ca^2+^ after treatment with thapsigargin in the absence of extracellular Ca^2+^ is likely due to Ca^2+^ extrusion via PMCA, since Ca^2+^ uptake into the ER via SERCA and alterations in Ca^2+^ influx are precluded under these conditions. Also, the low resting [Ca^2+^]_i_ can be explained by the strong expression of PMCA1 and 4. However, we cannot fully exclude a role for SPCA. The mRNA data show that ATP2C1 (encoding SPCA1), the prevailing ATP2C isoform in EA.hy926 cells, was not altered in SphK1-KD2 cells. Nevertheless, protein levels of these pumps may be regulated independently of mRNA. Furthermore, besides PMCA, another mechanism for extrusion of Ca^2+^ across the plasma membrane is provided by the Na^+^/Ca^2+^ exchanger. This protein is particularly important for removal of high [Ca^2+^]_i_ after depolarization of excitable cells such as muscle and nerve cells [46]. However, with its relatively low affinity for Ca^2+^, the Na^+^/Ca^2+^ exchanger is unlikely to reduce resting [Ca^2+^]_i_ levels below 100 nM in EA.hy926 SphK1 knockdown cells. It is rather PMCA, with its generally high Ca^2+^ affinity, which maintains the low resting [Ca^2+^]_i_ [46]. Thus, the decrease in resting [Ca^2+^]_i_ in SphK1-KD cells points towards the observed strong PMCA1 upregulation as underlying mechanism.

As a consequence of PMCA upregulation, two main mechanisms may contribute to the observed reduction of agonist-induced [Ca^2+^]_i_ increases in SphK1-KD1 cells: again, the rapid clearing of cytosolic Ca^2+^, and a reduced storage of Ca^2+^ in the ER. It is not very likely that the rapid peak responses to GPCR agonists, which were reached within 3–5 s, were that much decreased by PMCA activity as observed. Therefore, we conclude that there was less Ca^2+^ stored in the ER, potentially as a consequence of the reduced basal [Ca^2+^]_i_. Thapsigargin-induced [Ca^2+^]_i_ increases would then be attenuated by both enhanced clearing and reduced storage of Ca^2+^. However, we cannot exclude that SERCA activity was decreased and/or IP_3_ receptor sensitivity was altered in SphK1-depleted cells. The mRNA data show that the mainly expressed ATP2A2 (encoding SERCA2) was unaltered, but the less abundant ATP2A3 (SERCA3) was upregulated. Importantly, SERCA activity is regulated by many factors even in the absence of alterations in protein expression [45]. To address the above-mentioned questions, expression and activity of SERCA isoforms and IP_3_ receptors would have to be analyzed. However, in the light of the massive PMCA1 upregulation, we consider this as beyond the scope of the present study.

The activity of PMCA isoforms is differentially regulated by interactions with diverse proteins and lipids (for review, see [47, 55]). For example, PMCA activity was reduced and [Ca^2+^]_i_ was elevated in hippocampal neurons of mice with knockout of acid sphingomyelinase, while PMCA expression was not altered [33]. Furthermore, erythrocyte PMCA was activated by ceramide and inhibited by sphingosine [8, 44]; however, both ceramides and sphingosine d18:0 were decreased in SphK1-KD2 cells. Beyond these effects, our data show an upregulation of PMCA1 and PMCA4 protein expression in EA.hy926 SphK1-KD2 cells. Of note, PMCA1 was transcriptionally induced, with increase in both mRNA and protein, while PMCA4 was upregulated only on protein level with unaltered mRNA. In this regard, it might be important that basigin was markedly upregulated in SphK1-KD2 cells, both on mRNA and protein level. Basigin, and also the related neuroplastin, associates with PMCA in tetrameric complexes composed of two PMCA and two basigin or neuroplastin molecules [37, 13]. Basigin is a multifunctional transmembrane glycoprotein with two or three extracellular immunoglobulin domains that interacts with a wide number of proteins [28, 21]. By these interactions, which involve basigin’s transmembrane region, it stabilizes the interacting proteins, protects them from degradation, and facilitates their plasma membrane localization [21]. Therefore, it is reasonable to assume that the upregulation of basigin stabilized PMCA4 protein and enhanced its expression in the absence of ATP2B4 mRNA upregulation. It is furthermore likely that also the high PMCA1 protein expression was in part due to stabilization by basigin.

It remains the question how the transcription of PMCA1 and basigin was enhanced in SphK1-KD2 cells. Our data suggest that this occurred via altered histone acetylation, since H3K9 acetylation was enhanced in SphK1-KD2, and the HDAC inhibitor, vorinostat, increased transcription of ATP2B1 and basigin. It is already known that PMCA transcription can be induced by application of HDAC inhibitors such as vorinostat, also known as SAHA [18, 33]. Of note, by increasing PMCA expression, vorinostat counteracted sphingomyelin-induced PMCA inhibition in acid sphingomyelinase-deficient neurons [33]. Altered histone acetylation induced by S1P metabolism/intracellular S1P was first shown for nuclear SphK2 that provided S1P for direct binding to and inhibition of HDAC1 and HDAC2 [16]. Since then, a functional role for SphK2-dependent histone acetylation has been demonstrated in various conditions, for example, in the pathogenesis of pulmonary hypertension [35], or in kidney fibrosis [20]. Enhanced histone acetylation was also observed after depletion of Sgpl1, for example, in embryonic fibroblasts from Sgpl1 knockout mice [19], or in hippocampus, cortex and primary astrocytes of mice with nestin-Cre-dependent deletion of Sgpl1 [54]. These studies suggested that nuclear S1P, produced by SphK2 or accumulated in Sgpl1 deficiency, directly inhibited class I HDACs. However, SphK1-KD2 cells had very low concentrations of S1P. Although it might be possible that upregulation of SphK2 in SphK1-KD2 cells led to a local formation of S1P in the nucleus, not detectable in global cellular S1P measurements, we suggest that there is rather a different mechanism acting in these cells. Recent work has shown that the product of Sgpl1, hexadecenal, is a direct HDAC inhibitor [11]. Hexadecenal is a highly reactive molecule known to form protein adducts (see, e.g., [38]) and was shown to react with HDAC1 [11]. In our work on EA.hy926 cells with SphK1 knockdown, we did not address hexadecenal as the potential mediator of the observed histone acetylation. Yet, both SphK2 and SGPL1 were upregulated in SphK1-KD2, and the depletion of S1P, sphingosine, ceramides, and certain glycosphingolipids suggests a high metabolic flux via the two enzymes. Obviously, this will lead to enhanced formation of hexadecenal, at least transiently. We therefore suggest that this mechanism caused HDAC inhibition in SphK1-KD2 cells despite reduced levels of S1P.

EA.hy926 cells have previously been used to show that treatment with NO donors increased SphK1 expression, and knockdown of SphK1 attenuated NO-dependent migration and tube formation in these cells [39]. Whether the upregulation of PMCA1 or PMCA4 observed here contributed to this phenotype remains unclear, since these two PMCA isoforms had opposite effects on endothelial cell migration and angiogenesis: PMCA1 silencing in HUVEC increased [Ca^2+^]_i_ and enhanced endothelial NO synthase activity [26], but decreased viability, migration, and tube formation in response to VEGF [29]. In contrast, endothelial cells from PMCA4 knockout mice displayed enhanced migration, while overexpression of PMCA4 suppressed VEGF-induced migration and angiogenesis [2].

Taken together, our data for the first time demonstrate a regulation of PMCA expression by cellular S1P metabolism. SphK1 knockdown in EA.hy926 cells caused complex alterations in cellular sphingolipid homeostasis, which finally altered histone acetylation and thereby induced the expression of PMCA1 and its accessory protein, basigin. Although targeting SphK1 might be a promising approach for compensating PMCA1 loss-of-function mutations by upregulating the protein, we assume that the observed sphingolipid alterations in EA.hy926 cells are probably cell-type specific. Further work is required to understand fully how S1P metabolism targets PMCA and regulates Ca^2+^ homeostasis in different cell types.

## Data Availability

Data is provided within the manuscript.
